# Tractography-Enhanced Biopsy of Central Core Motor Eloquent Tumours: A Simulation-Based Study

**DOI:** 10.3390/jpm13030467

**Published:** 2023-03-03

**Authors:** Harishchandra Lalgudi Srinivasan, Jose Pedro Lavrador, Kantharuby Tambirajoo, Graeme Pang, Sabina Patel, Richard Gullan, Francesco Vergani, Ranjeev Bhangoo, Jonathan Shapey, Ahilan Kailaya Vasan, Keyoumars Ashkan

**Affiliations:** 1 Department of Neurosurgery, King’s College Hospital, London SE5 9RS, UK; 2 King’s NeuroLab, King’s College Hospital, London WC2R 2LS, UK; 3 Department of Surgical Intervention and Engineering, School of Biomedical Engineering and Imaging Sciences, King’s College London, London WC2R 2LS, UK

**Keywords:** tractography, stereotactic biopsy, neuronavigation, motor eloquent tumours

## Abstract

Safe Trajectory planning for navigation guided biopsy (nBx) of motor eloquent tumours (METs) is important to minimise neurological morbidity. Preliminary clinical data suggest that visualisation of the corticospinal tract (CST) and its relation to the tumour may aid in planning a safe trajectory. In this article we assess the impact of tractography in nBx planning in a simulation-based exercise. This single centre cross-sectional study was performed in March 2021 including 10 patients with METs divided into 2 groups: (1) tractography enhanced group (T-nBx; *n* = 5; CST merged with volumetric MRI); (2) anatomy-based group (A-nBx; *n* = 5; volumetric MRI only). A biopsy target was chosen on each tumour. Volunteer neurosurgical trainees had to plan a suitable biopsy trajectory on a Stealth S8^®^ workstation for all patients in a single session. A trajectory safety index (TSI) was devised for each trajectory. Data collection and analysis included a comparison of trajectory planning time, trajectory/lobe changes and TSI. A total of 190 trajectories were analysed based on participation from 19 trainees. Mean trajectory planning time for the entire cohort was 225.1 ± 21.97 s. T-nBx required shorter time for planning (*p* = 0.01). Mean trajectory changes and lobe changes made per biopsy were 3.28 ± 0.29 and 0.45 ± 0.08, respectively. T-nBx required fewer trajectory/lobe changes (*p* = 0.01). TSI was better in the presence of tractography than A-nBx (*p* = 0.04). Neurosurgical experience of trainees had no significant impact on the measured parameters despite adjusted analysis. Irrespective of the level of neurosurgical training, surgical planning of navigation guided biopsy for METs may be achieved in less time with a safer trajectory if tractography imaging is available.

## 1. Introduction

Motor eloquent tumours (METs) are supratentorial lesions located in proximity to the corticospinal tract (CST) or Rolandic cortex [1,2,3]. METs pose a higher risk for long-term post-resection neurological deficit and a stereotactic biopsy may be the first step before planning a more definitive management [1,4]. However, navigation-based biopsy (nBx) may also lead to permanent neurological morbidity in 2.5–5% of patients [4,5]. A post-operative neurological deficit alters the performance status of a patient, reducing overall survival and rendering them ineligible for aggressive adjuvant treatment [6,7]. Many biopsy-associated neurological deficits are related to haemorrhage or oedema near a Rolandic cortex or CST [5,8]. If the proximity of a trajectory to an eloquent structure is identified pre-operatively, the likelihood of a complication could potentially be averted. 

A tumour may interact with white matter tracts in different ways, including deviation, destruction, or infiltration [9]. Hence, it is difficult to predict the spatial relationship between a tumour and white matter tracts based on anatomical landmarks alone [4,10,11]. Tractography is a valuable imaging modality that provides in-vivo visualization of the white matter tracts [12]. Pre-operative anatomical dissection of these tracts may help the surgeon in deciding the best trajectory. Few studies have analysed the influence of tractography on image guided biopsy [4,13]. Preliminary data from these studies suggest that tractography enhanced nBx may decrease morbidity by influencing a surgeon to modify the trajectory based on the interaction between white matter fibres and the tumour [4,13]. However, tractography has its own inherent issues, including different protocols to image the tracts (diffusion tensor model vs constrained spherical model [14]); and different tracking algorithms (deterministic vs probabilistic approach [12,15]). Each of these techniques has its own pros and cons, which may limit the utility of a tractography [12,15].

Neurosurgical trainees are regularly exposed to navigation-based procedures and performing a surgical biopsy is a required core competency of training. Recently, virtual reality-based training has gained popularity to improve neurosurgical education, particularly in so-called high-risk pathologies [16]. Simulation training has an applicability beyond the planning that could directly impact in the actual surgical performance in theatre [17]. In this study, with the help of simulation training we assessed the impact of using tractography-enhanced images in the surgical planning of nBx in METs. The impact of tractography-enhanced-nBx for METs upon patient safety was inferred from data in the clinical literature.

## 2. Methods

A single centre cross-sectional study was conducted in March 2021. The study was registered as an audit with and approved by the local department (Neuro-oncology service, Department of Neurosurgery, King’s College Hospital). Tumours arising from or within motor cortex or within/adjacent to the corticospinal tract (<10 mm) were considered as motor eloquent tumours (METs) [3]. The pre-operative images of 10 patients with METs in the central core (based on Ribas et al. [18]) were selected. All patients provided informed consent, and all related data were anonymized. In 5 patients, only T1-Gadolinium image was used—Anatomy-based nBx group (A-nBx). In the other 5 patients, preoperatively dissected CST was merged with the T1-Gadolinium image—Tractography-enhanced nBx group (T-nBx). A region-of-interest (ROI) approach was used to generate the tractography: one ROI was placed over the ipsilateral M1 and the other ROI in the brainstem. All the dissections were performed by a senior neurosurgeon (J.P.L.) using Stealth Viz Medtronic^©^ Software. The authors selected a target to biopsy for each patient and ensured the target was within the boundaries of the central core [18].

Neurosurgical trainees voluntarily enrolled themselves for the simulation exercise. If a trainee had already been involved in biopsy planning of any of the METs in the past, they were excluded. Volunteers were asked to plan a biopsy trajectory for each patient using a Stealth S8^®^ Workstation. Prior to the start of the exercise, all volunteers were given a brief explanation on what to expect. They were also given a demonstration on how to plan on a Stealth S8^®^ Workstation. Planning had to be performed on all 10 tumours in a single session. Patients were presented in an alternating fashion from both groups and same order was retained for all volunteers. We neither influenced nor guided their planning exercise. However, during the planning exercise one of the authors would observe the trainee. At the end of the exercise, a debrief session was held with the trainee as a part of the learning process.

Data collected included: experience of trainee, length of trajectory, trajectory planning time, number of trajectory/lobe changes for each tumour, proximity of trajectory to vessels, proximity to midline, proximity to CST (only tractography group); and whether a trajectory transgressed a sulcus, eloquent cortex (M1), or ventricle.

**Figure 1:**
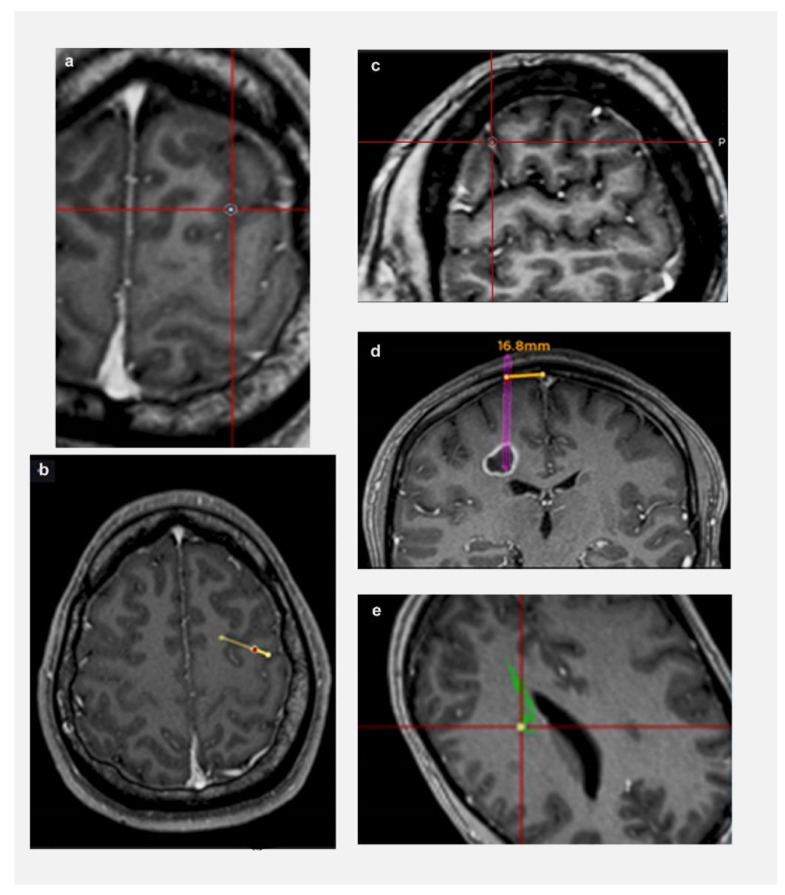
Defining relationship to critical neurovascular structures. (**a**) Sulcal transgression of trajectory; (**b**) Transgression of eloquent cortex by trajectory (arrow represents central sulcus); (**c**) Proximity to vessel—Zone 1 (the rim represents 3 mm safety margin); (**d**) Proximity to midline—trajectory is 16.8 mm from midline (Zone 1 < 20 mm from midline); (**e**) Proximity to CST—Zone 1 (the rim represents 3 mm safety margin).

The following parameters were defined prior to data collection (shown in Figure 1): (a) Length: from target to entry point on cortical surface; (b) Trajectory planning time: from the time a trainee is presented with a tumour target until they have finalised their trajectory and move on to next tumour; (c) Trajectory changes: number of times a trainee modified their trajectory for each target; (d) Lobe changes: number of times a trainee modified their lobe of entry (frontal/ parietal/ temporal/ occipital—based on standard anatomical landmarks) for each target; (e) Proximity to vessels: distance from a vessel: <3 mm (Zone 1); >3 mm (Zone 2); (c) Proximity to CST: distance from CST: <3 mm (Zone 1); >3 mm (Zone 2); and (f) Proximity to midline: distance from midline: <20 mm (Zone 1); >20 mm (Zone 2).

“Zone 1” was defined as a high risk trajectory zone for vascular injury or neurological deficit based on the existing literature [4,19,20]. Similarly, a trajectory transgressing a sulcus, ventricle, or eloquent cortex, had a higher risk for vascular or neurological injury [21]. Considering all these factors, a “Trajectory safety index” (TSI) was analysed for each trajectory. One point was assigned for each of the following parameters: transgressing sulcus; eloquent cortex; zone 1 vessels; zone 1 midline. Based on our trajectory safety index, higher the points, the higher the risk of a vascular/neurological injury. Therefore, the safety of the trajectory was assessed by an objective index and not by the potentially biased opinion of an expert in the field, providing comparable data among individuals and institutions.

Statistical analysis was performed using STATA 13.1 Software^©^. Univariate and Multivariate logistic regression analysis was performed to assess the impact of neurosurgical experience and tractography on trajectory planning time and safety index. A P value of 0.05 was considered significant. A uniform manifold approximation and projection using BioVinci Software^©^ was used to perform graph-based clustering analysis considering the number of trajectory and lobe changes, availability of corticospinal tract tractography and the normalized time used to define the final biopsy trajectory [22]. 

## 3. Results

A total of 190 trajectories were analysed following participation by 19 neurosurgical trainees (4 female and 15 males). Five trainees were in their initial stages of training (ST1/2), and seven each from intermediate (ST3/4/5) and advanced stages (ST6/7/8) of training [23]. Average experience in neurosurgery of the entire cohort was 4.10 years. 

### 3.1. Trajectory Planning Time

**Figure 2:**
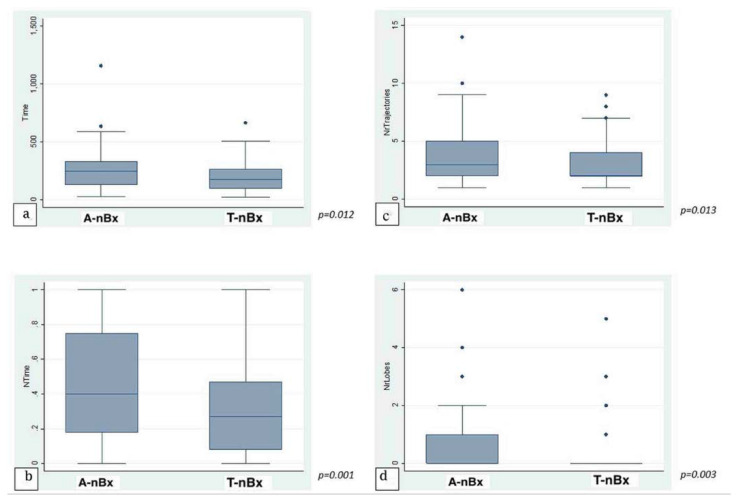
Trajectory enhanced nBx vs Anatomy-based nBx group—box-plot analysis. (**a**) Absolute time comparison (*Y* axis—time in seconds); (**b**) Normalized time comparison; (**c**) Trajectory changes; (**d**) Lobe changes. (NrTrajectories—Number of trajectory changes; NrLobes—Number of Lobe changes).

**Figure 3:**
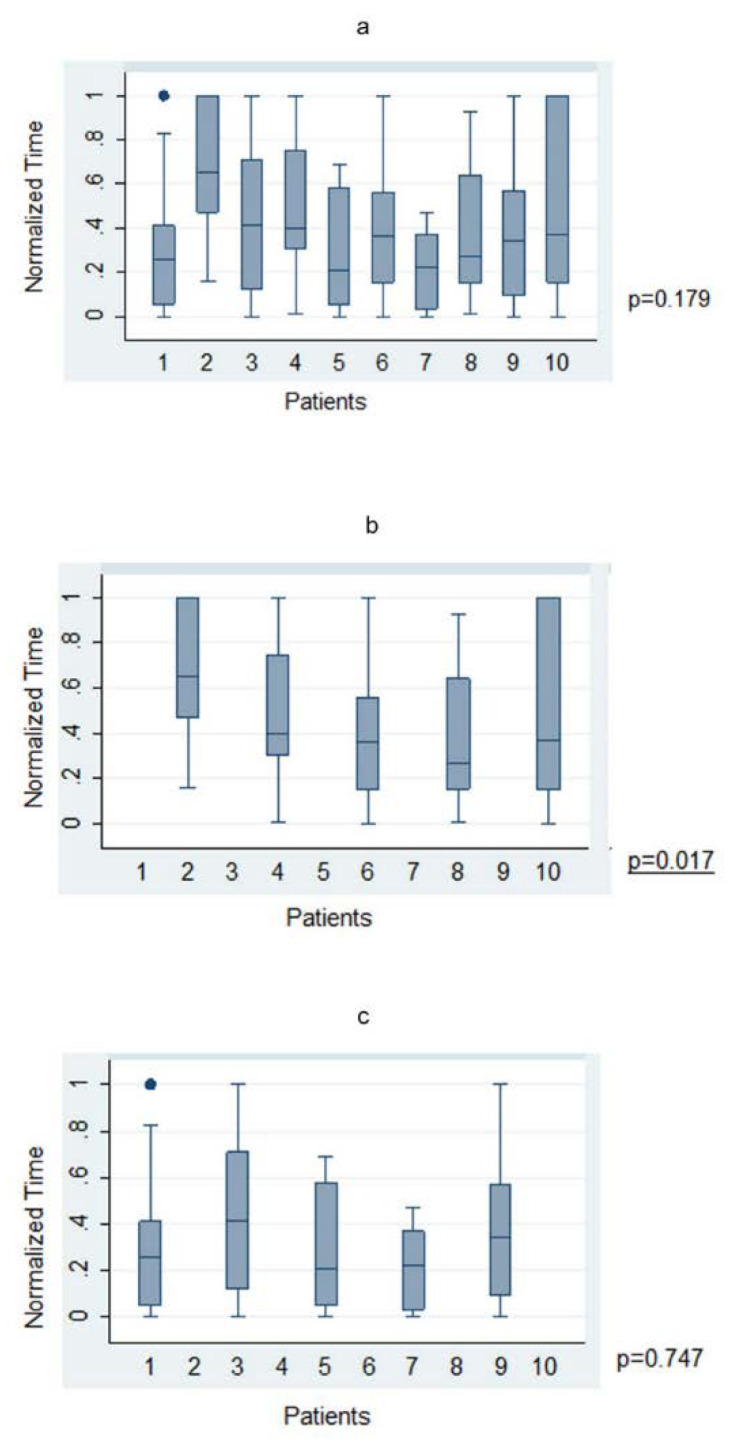
Trajectory planning time—Trajectory-based nBx (T-nBx) vs. Anatomy-based nBx group (A-nBx)-box-plot analysis; (**a**) Trajectory planning time from exercise 1 to exercise 10 for entire cohort; (**b**) Trajectory planning time from exercise 1 to exercise 10 for A-nBx group; (**c**) Trajectory planning time from exercise 1 to exercise 10 for T-nBx group box plot analysis.

The average time taken to plan a trajectory for a given target was under 4 min (225.1 ± 21.97 s; range 23–1158 s). There was no significant statistical correlation between level of training and time taken to plan a trajectory across the whole cohort in both absolute (*p* = 0.77) and normalized (*p* = 0.53) times. When anatomy (A-nBx) and tractography-enhanced (T-nBx) groups were compared, the absolute and normalized planning time was significantly higher in the A-nBx group (252.8 ± 16.8 s vs. 197.5 ± 12.9 s, *p* = 0.01; 0.48 ± 0.03 vs. 0.33 ± 0.03, *p* = 0.001) (Table 1). These results when adjusted to the years of neurosurgical training, remained unchanged (absolute time *p* = 0.01; normalized time *p* = 0.001) (shown in Figure 2). The impact of the learning curve during the exercise was assessed comparing the times taken to perform the planning across the whole simulation. No difference was identified across the whole cohort of patients for both absolute (*p* = 0.74) and normalized (*p* = 0.50) times. However, when both subgroups were considered, a statistically significant reduction in the planning time was observed across the A-nBx subgroup (*p* = 0.02) but not the T-nBx subgroup (*p* = 0.75) (shown in Figure 3).

### 3.2. Trajectory/Lobe Changes

On average, 3.28 ± 0.29 trajectory changes and 0.45 ± 0.08 lobe changes were made for every plan across the whole cohort. In A-nBx, significantly more trajectory (3.66 ± 0.24 versus 2.89 ± 0.17, *p* = 0.01) and lobe changes (0.66 ± 0.11 versus 0.23 ± 0.08, *p* = 0.003) were performed when compared with the T-nBx (Table 1) (shown in Figure 2). This analysis when adjusted for the years of neurosurgical experience, remained unchanged (number of trajectory changes, *p* = 0.01; number of lobe changes, *p* = 0.003).

### 3.3. Trajectory Safety Index

**Figure 4:**
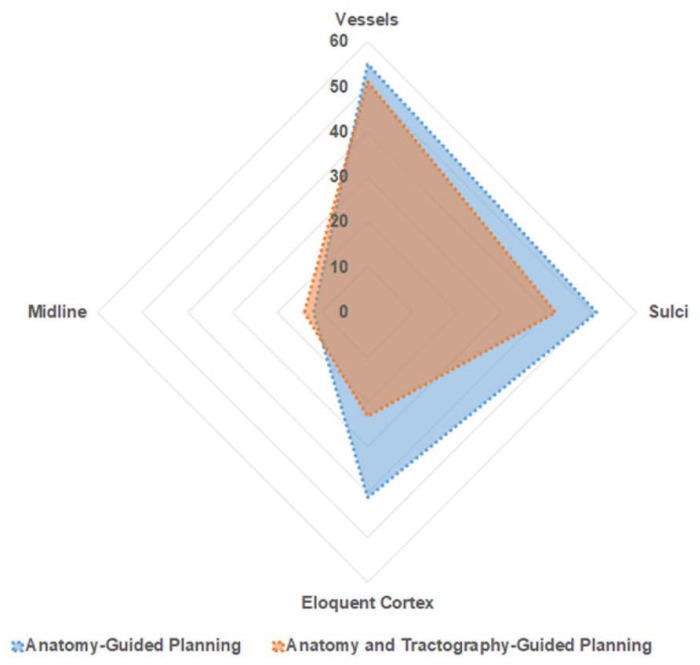
Trajectory Safety Index—Area plot—explaining distribution of trajectories in relation to proximity to neurovascular structure. Blue—represents A-nBx group; Red—represents T-nBx group.

The mean length of the planned trajectories across the entire cohort was 59.24 ± 8.3 mm. Trajectories performed on the T-nBx group had significantly shorter trajectories (56.10 ± 1.25 mm vs. 62.37 ± 1.0 mm, *p* < 0.001) (shown in Figure 4). These results remained stable when the analysis was adjusted for the years of neurosurgical experience (*p* < 0.001). 

Among the entire cohort of 190 trajectories, 106 trajectories passed through the high-risk zone (zone 1) for a vessel. There was no significant difference between the two subgroups (A-nBx 55; T-nBx 51; *p* = 0.56). Zone 1 for midline was traversed by 26 trajectories and there was no difference between the 2 subgroups (A-nBx 12; T-nBx 14; *p* = 0.65). In the T-nBx subgroup, zone 1 of a CST was traversed by 14 trajectories and the incidence did not vary based on experience (*p* = 0.15). A sulcal transgression was observed in 93 trajectories with no significant difference between the 2 subgroups (A-nBX 51; T-nBx 42; *p* = 0.19). The ventricle was transgressed by a single trajectory in the A-nBx group. Motor eloquent cortex was transgressed by 64 trajectories but significantly fewer transgressions when tractography enhanced images were utilized (A-nBx 41; T-nBx 23; *p* = 0.01). Across each of the safety index parameter, the results remained unchanged when adjusted for years of neurosurgical experience as illustrated in Table 2.

As described in the methods section, a trajectory safety index model was elaborated. The safety of a trajectory did not correlate with trainee experience (*p* = 0.19). This may be related to planning with additional data (tractography) on a virtual platform. However, trainee experience may be significant when performing a navigation guided biopsy which involves more than just planning a trajectory. Tractography-enhanced trajectories had a better safety index (mean safety index: 1.37 ± 0.11 versus 1.70 ± 0.12, *p* = 0.04).

### 3.4. Graph-Based Clustering

**Figure 5:**
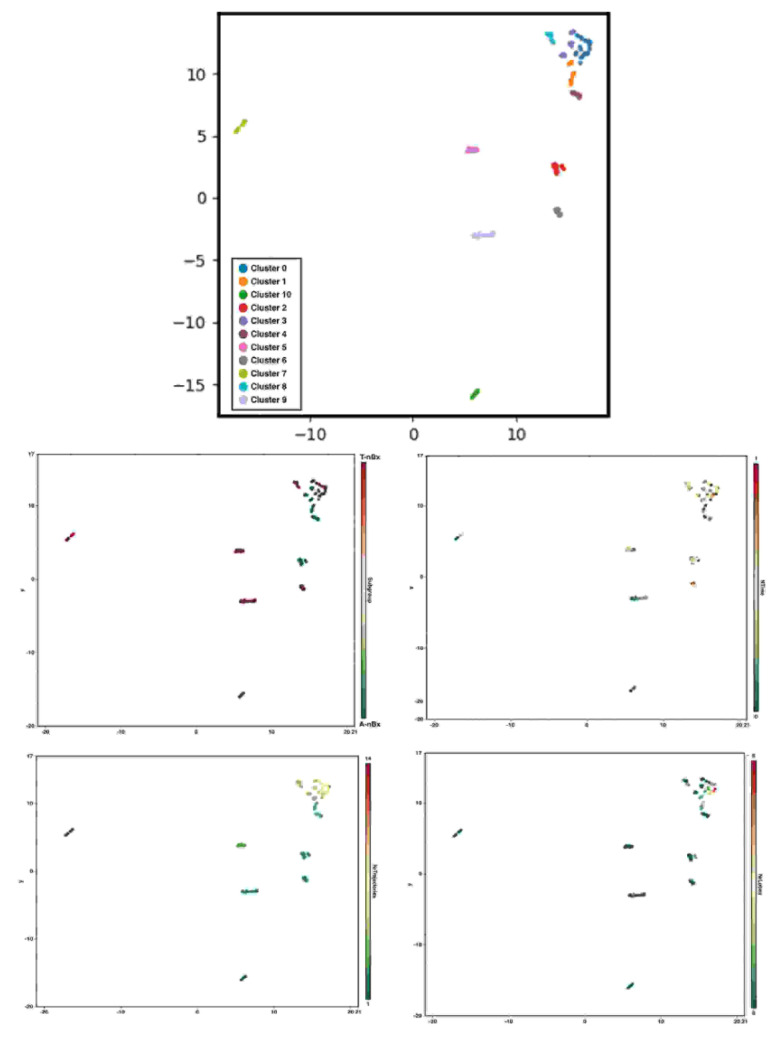
Graph-based clustering and subcomponent analysis (CST tractography availability, normalized time to perform the planning, number of trajectory and lobe changes) according to uniform manifold approximation and projection.

Eleven clusters of nBx planning were identified in this cohort based on the number of trajectory and lobe changes, the availability of CST tractography and the normalized time to perform the planning (shown in Figure 5). Cluster 1 mimicked A-nBx planning (more trajectory and lobe changes along with prolonged trajectory planning time) whereas cluster 9 was like T-nBx planning (fewer trajectory and lobe changes with shorter normalized time to complete the planning). Cluster 0 included nBx plans with the most trajectory, lobe changes and prolonged normalized planning time when compared with the others. Other clusters occupy a more intermediate position. For example, cluster 2 includes A-nBx planning, but with fewer trajectory and lobe changes whereas cluster 8 includes T-nBx planning with prolonged planning time and more trajectory changes. Each of the 11 clusters represents a unique trainee-tumour interaction profile based on subtle differences between various components analysed. 

## 4. Discussion

To the best of our knowledge, this study is the largest series (190 trajectories) analysing the role of tractography in nBx for central core METs [4,13] in a simulation setting. Information provided by the tractography does influence biopsy planning of the METs resulting in shorter planning time, fewer trajectory/ lobe changes and safer trajectories. 

### 4.1. Tractography-Enhanced Planning Reduces Trajectory Planning Time

It had been anticipated that trainees would take longer to plan biopsy trajectories in the T-nBx group, when trying to navigate around the CST to reach the target. However, planning was faster in the T-nBx group (*p* = 0.001; shown in Figure 2). We suggest that the availability of tractography data simplified planning by highlighting the critical structures to be avoided. Interaction between the CST and a tumour is unpredictable in the absence of tractography [10,11,24]. Hence, without tractography, a trainee may be unsure if their trajectory avoids the CST, thereby prolonging the planning time. 

As trainees progressed across the exercise, trajectory planning time significantly shortened for the A-nBx group (shown in Figure 3). This highlights a learning curve involved with anatomy-based planning. An improved capability to predict (or realising the inability to predict) the course of CST may be responsible for gradual shortening of planning time. However, with tractography the difference in biopsy planning time related to the seniority of neurosurgical training was insignificant. The uncertainty in the young neurosurgical trainees caused by the absence of CST dissection was overcome by the experience in the case of senior neurosurgical trainees used to performing A-nBx. When the CST is dissected and presented in the simulation, the experience becomes less significant for the outcome and the differences become non-significant. Conducting a similar exercise with more experienced neurosurgeons (consultants) and superimposition of tracts post-planning may be required to fully understand the learning curve. 

### 4.2. Tractography-Enhanced Planning and Changes in the Trajectories/Lobes

More trajectory changes were expected in the tractography group to navigate around the CST. However, tractography-enhanced planning had significantly fewer changes than in the A-nBx group (*p* = 0.01). The dilemma over the course of CST in A-nBx may be responsible for the increased trajectory/lobe changes [9,10,11]. Tractography explicitly demonstrates the relation between CST and a tumour, thereby being responsible for a more certain plan with fewer changes. 

### 4.3. Tractography-Enhanced Planning Aids in Safe Trajectory Planning

**Figure 6:**
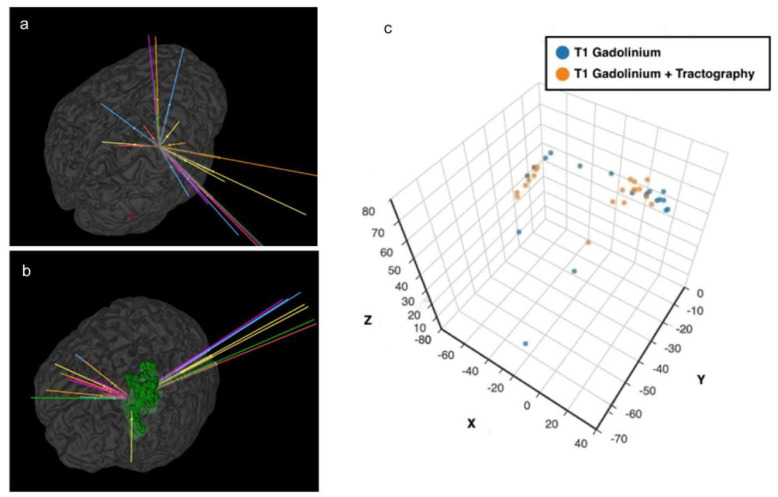
Proximity to midline and tractography. (**a**) Anatomy-based nBx—trajectories are scattered all over the hemisphere; (**b**) Tractography enhanced nBx—trajectories are pushed closer to midline in an intent to avoid CST; (**c**) Spatial representation of the cortical entry points of the different strategies based on X, Y and Z coordinates after definition of the line connecting the anterior and posterior commissures (AC-PC) and the middle commissural point (MCP).

A safe trajectory depends on several factors including but not limited to tumour location, transgressing a sulcus, vessel or a ventricle, length of trajectory and angle of entry across the skull (obliquity) [5,8,20,21,25]. The closer a trajectory is to a critical neurovascular structure, the higher the incidence of morbidity [4,5,19,21]. Trajectories planned with tractography had a better safety index (*p* = 0.04) than the A-nBx group. This was predominantly influenced by shorter trajectory lengths (*p* < 0.001) and fewer transgressions into eloquent cortex (*p* = 0.01). However, with tractography, trajectories were pushed closer to the midline, possibly related to lateral displacement of CST by all tumours, (shown in Figure 6). This may be related to the location of tumours we selected for this exercise (a sampling bias). 

In the tractography group, 14.7% of trajectories were passing close to the CST (zone 1). With the anatomy guided biopsies, there is a higher risk of transgressing the CST unknowingly due to the unpredictable interaction between the tumour and the CST. There are only 2 studies in the literature which directly compare the influence of tractography in stereotactic biopsy [4,13]. Around 36–58% of surgeons modified their trajectory after superimposing the tracts on their anatomy-guided planning [4,13]. Hence the risk of transgressing CST is presumed to be higher when tractography is unavailable, thereby increasing the risk for neurological morbidity (shown in Figure 4).

It is important that virtual reality and simulation-based research is focused on translational skills that could go beyond a technical exercise but have direct application in the neurosurgical practice [17]. Clustering analysis provides an interesting overview of different trainee-tumour interactions based upon the individual planning strategies. Apart from the cluster-type identified for both A-nBx (cluster 1) and T-nBx (cluster 9) there are different intermediate clusters with small changes depending on the interaction between the components analysed. We believe educational strategies should be put in place according to the clustering profile of the neurosurgical trainees. For example, a trainee from cluster 8 (T-nBx with multiple trajectory changes and prolonged planning time) is probably not used to planning with tractography and may need to focus on planning more nBx with tractography. As cluster analysis summarizes more comprehensive information, it may provide a more accurate assessment and tailored approach to match trainees’ needs.

### 4.4. Tractography—Reliability Limitation

In this study, robust dissection of all CST was performed by a single experienced neurosurgeon to provide uniformity. However, the accuracy and precision of the reconstructed tracts may not be reliable. There are several steps involved in drawing the tracts -image acquisition, local reconstruction of tracts, and fibre tracking voxel by voxel [26]. At each step, there are pitfalls and despite correction the reliability of reconstructed tracts is still limited [26]. These inherent issues with tractography (cost, user-dependence in drawing tracts, fibre-tracking program, brain shift) were not considered in this study [12,14,15,24]. However, the purpose of this study was to understand the change in biopsy plan when a surgeon is aware of the course of tracts. 

Future studies should analyse the impact of other imaging modalities (fMRI; nTMS etc) and consider the influence of other white matter tracts (language and visual function) on image guided biopsy. Meticulous planning with all the imaging data may help in thwarting complications. However, whether the theoretical advantage provided by these additional investigations translates into preventing a morbidity needs a more detailed clinical study.

### 4.5. Limitations and Future Work

A conceptional limitation of this study is the surrogate assessment of the safety of a needle biopsy procedure based on a range of variables, supported by the existing literature [4,5,21,27], rather than by actual patient outcomes. We believe this is justified since there is no equipoise to prospectively submit patients to potentially higher risk procedures to evaluate clinical outcomes. Comparing a prospective cohort of patients undergoing tractography based biopsy with historical controls in whom tractography was not utilized is an option but will be prone to the usual biases of retrospective data collection and unmatched controls. Therefore, a simulation-based study, such as ours, evaluating various aspects of the targeting techniques can provide valuable surrogate data on safety. 

Simulation-based studies have been shown to be extremely beneficial to neurosurgical training, and accurately reflect clinical practice [28]. The aim of this study was to assess safe trajectory planning in the performance of neurosurgical trainees in a virtual setting. Results may have been different if experienced neurosurgeons had been a part of this study; however, by including trainees across all stages of training, including those close to completion of training we believe that we addressed this limitation in methodology. Planning 10 trajectories in a single session may have induced mental fatigue and may have contributed to poor planning. We used a small sample size of 10 tumours, but our data of 190 trajectories was a significant number to analyse the role of tractography. We had a homogenous tumour group (central core METs) but chose not to provide the same tumour in both groups (unlike other studies [4,13]) to negate the possibility of memory bias during planning. CST was the only tract analysed in this study. Robust dissection of all CST was performed by a single experienced neurosurgeon to provide uniformity. The inherent issues with tractography (cost, user-dependence in drawing tracts, fibre-tracking program, brain shift [12,14,15,24]) were not considered in this study. Future studies should analyse the impact of other imaging modalities (fMRI; nTMS etc), consider the influence of other white matter tracts (language and visual function) on image guided biopsy and provide a large volume of data that could bring new opportunities for artificial intelligence-based models. Meticulous planning with all the imaging data may help in thwarting complications. 

## 5. Conclusions

Navigated biopsy of motor eloquent tumours of the central core has a potential significant morbidity. This study provides data suggesting that tractography does not provide redundant information in biopsy planning but that it can make a difference to the patient and the surgeon during biopsy planning. Therefore, non-simulation studies using systematic tractography data should be performed in the future to provide data supporting this approach.

## Figures and Tables

**Table 1:** Univariate Statistical Analysis of trajectory planning time (absolute and normalized), trajectory and lobe changes in the whole cohort and comparing both A-nBx and T-nBx groups.

	Whole Cohort (n-190)	T-nBx Group (n-95)	A-nBx Group (n-95)	*p* Value	*p* Value (after Adjusting for Neurosurgical Experience)
Trajectory Planning Time (s) Absolute Values	225.1 ± 21.97	197.5 ± 12.9	252.8 ± 16.8	0.012	0.012
Trajectory Planning Time (s) Normalization	0.40 ± 0.02	0.33 ± 0.03	0.48 ± 0.03	0.001	0.001
Trajectory Changes	3.28 ± 0.29	2.89 ± 0.17	3.66 ± 0.24	0.013	0.013
Lobe Changes	0.45 ± 0.08	0.23 ± 0.08	0.66 ± 0.11	0.003	0.003

T-nBx: Trajectory enhanced navigation guided biopsy; A-nBx: Anatomy based navigation guided biopsy.

**Table 2:** Trajectory Safety index Analysis.

Safety Parameter	Overall Incidence n-190 (%)	T-nBx Group (n-95)	A-nBx Group (n-95)	*p* Value	*p* Value (after Adjusting for Neurosurgical Experience)
Vessel	106 (55.79)	51	55	0.559	0.558
Sulcus	93 (48.95)	42	51	0.192	0.190
Eloquent cortex	64 (33.86)	23	41	0.007	0.007
Ventricle	1 (0.53)	0	1	-	-
Midline	26 (13.76)	14	12	0.652	0.655
CST (only T-nBx group; n-95)	14 (14.74)	14	0	-	-
Length of trajectory (mm)	59.24 ± 8.3	54.54 ± 8.3	62.37 ± 5.87	<0.001	<0.001

T-nBx: Trajectory enhanced navigation guided biopsy; A-nBx: Anatomy based navigation guided biopsy; CST: Corticospinal tract.

## Data Availability

All data generated or analysed during this study are included in this article. Further enquiries can be directed to the corresponding author.
